# Extracellular HSP90α Interacts With ER Stress to Promote Fibroblasts Activation Through PI3K/AKT Pathway in Pulmonary Fibrosis

**DOI:** 10.3389/fphar.2021.708462

**Published:** 2021-08-23

**Authors:** Jinming Zhang, Wenshan Zhong, Yuanyuan Liu, Weimou Chen, Ye Lu, Zhaojin Zeng, Yujie Qiao, Haohua Huang, Xuan Wan, Wei Li, Xiaojing Meng, Fei Zou, Shaoxi Cai, Hangming Dong

**Affiliations:** ^1^ Chronic Airways Diseases Laboratory, Department of Respiratory and Critical Care Medicine, Nanfang Hospital, Southern Medical University, Guangzhou, China; ^2^ Department of Dermatology and The Norris Comprehensive Cancer Centre, University of Southern California Keck Medical Centre, Los Angeles, CA, United States; ^3^ School of Public Health, Southern Medical University, Guangzhou, China

**Keywords:** extracellular Hsp90α, er stress, fibroblasts activation, PI3K/AKT, pulmonary fibrosis

## Abstract

Pulmonary fibrosis is characterized by alveolar epithelial cell injury, lung fibroblast proliferation, differentiation, and extracellular matrix (ECM) deposition. Our previous study indicated that extracellular HSP90α (eHSP90α) promotes pulmonary fibrosis by activating the MAPK signaling pathway. Thus, treatment with 1G6-D7 (a selective HSP90α monoclonal antibody) to antagonize eHSP90α could effectively ameliorate fibrosis. This study aimed to elucidate the mechanism underlying the effects of eHSP90α in pulmonary fibrosis by focusing on its link with endoplasmic reticulum (ER) stress. Our results showed that eHSP90α promoted lung fibroblast differentiation by activating ER stress. Treatment with the ER stress inhibitor tauroursodeoxycholate (TUDCA) or glucose-regulated protein 78 kDa (GRP78) depletion significantly abrogated the effect of eHSP90α on ER stress and fibroblast activation. In addition, eHSP90α induced ER stress in fibroblasts *via* the phosphoinositide-4,5-bisphosphate 3-kinase (PI3K)-protein kinase B (AKT) signaling pathway, which could be blocked by the PI3K/AKT inhibitor LY294002, and blockade of eHSP90α by 1G6-D7 markedly inhibited ER stress in the model, indicating preventive and therapeutic applications. Intriguingly, we observed that TUDCA effectively reduced the secretion of eHSP90α *in vitro* and *in vivo*. In conclusion, this study shows that the interaction between eHSP90α and ER stress plays a crucial role in pulmonary fibrosis, indicating a positive feedback in lung fibroblasts. Targeting eHSP90α and alleviating fibroblast ER stress may be promising therapeutic approaches for pulmonary fibrosis.

## Introduction

Pulmonary fibrosis is a chronic, progressive, fibrotic interstitial pulmonary disease of unknown origin that results in reduced exchange and impaired pulmonary function. To our knowledge, pulmonary fibrosis is one of the most forms of common interstitial pneumonia, presenting with a high morbidity rate and lacking effective therapies to improve the survival rate. Pirfenidone and nintedanib have been recently shown to have a moderate effect on disease progression. However, neither agent stops pulmonary fibrosis progression (Martinez et al., 2017; Richeldi et al., 2017). Therefore, it is essential to develop alternative therapeutic strategies for patients with PF. The pathological characteristics of pulmonary fibrosis include alveolar epithelial injury, aberrant fibroblast differentiation and proliferation, and excessive pro-fibrotic cytokine secretion (Wolters et al., 2014). Notably, with the stimulation of multiple pro-fibrotic cytokines, lung fibroblasts differentiate into myofibroblasts, leading to massive ECM accumulation and accelerated fibrosis progression (Kwon et al., 2018; Duan et al., 2019; Li et al., 2019). Therefore, fibroblasts/myofibroblasts play a central role in fibrosis formation, and suppression of fibroblast differentiation could be an important strategy to alleviate pulmonary fibrosis.

The endoplasmic reticulum (ER) plays a key role in cellular homeostasis and is extremely sensitive to various changes. Failure of the ER to fold and assemble proper protein architecture leads to accumulation of misfolded/unfolded proteins in the ER lumen, disturbing ER homeostasis and provoking ER stress. ER stress-associated proteins mainly include GRP78, activating transcription factor-6 (ATF6), and inositol-requiring enzyme-1α (IREα). The main function of these proteins is to expand the ER protein-folding capacity and reduce ER load. ER stress has been recently noted in various diseases, including cancer, asthma, and diabetes (Cubillos-Ruiz et al., 2017; Bhakta et al., 2018; Crookshank et al., 2018). For instance, multiple cancers have a sustained and abnormally high expression of ER-related proteins (Fernandez et al., 2000; Shuda et al., 2003; Carrasco et al., 2007). In addition, ER stress is also involved in lung fibrosis by regulating fibroblast proliferation, differentiation, and alveolar epithelial injury (Lee et al., 2020a; Borok et al., 2020). Treatment with the ER stress inhibitor 4-phenylbutyrate (4-PBA) or TUDCA could effectively attenuate pulmonary fibrosis (Hsu et al., 2017; Lee et al., 2020b). Therefore, further investigation of the molecular mechanisms underlying ER stress in pulmonary fibrosis is highly appreciated.

The levels of heat shock protein 90 (HSP90), one of the most abundant HSPs, have been reported to be elevated in IPF patients and experimental pulmonary fibrosis. Furthermore, HSP90 inhibition with 17-AAG or AUY-922 could help alleviate pulmonary fibrosis by blocking the transforming growth factor-β (TGF-β) signaling pathway (Colunga et al., 2020). Notably, HSP90 has been confirmed to be secreted from cells following multiple stresses such as hypoxia, reactive oxygen species and heat, and this secreted form is called eHSP90α. Emerging evidence indicates that eHSP90α is associated with tumor progression and wound healing (Li et al., 2012; Fan et al., 2019). In addition, we previously confirmed that eHSP90α promotes pulmonary fibrosis by activating the MAPK signaling pathway, and the use of the monoclonal antibody 1G6-D7 could effectively attenuate pulmonary fibrosis (Dong et al., 2017). As mentioned above, ER stress has a positive effect on the activation of lung fibroblasts in pulmonary fibrosis. However, the relationship between eHSP90α and ER stress in pulmonary fibrosis has not yet been completely clarified.

In this study, we examined the crosstalk between eHSP90α and ER stress in lung fibroblasts. The role of eHSP90α in the regulation of ER stress depends on activating the PI3K/AKT signaling pathway. We also confirmed ER stress mediated eHSP90α released in the pulmonary fibrosis.

## Materials and Methods

### Cell Culture

IMR90 cells were purchased from ATCC and cultured in EMEM medium supplemented with 10% fetal bovine serum (PAN, German) in an atmosphere of 5% CO2. When the cells were 80–90% confluent, they were stimulated with recombinant TGF-β1 (R&D Systems, United States) with or without TUDCA (MCE, United States) for another 24 h. Before stimulation with human recombinant Hsp90α (hrHsp90α; Stress Marq Biosciences, British Columbia), the cells were pretreated with LY294002 (MCE, United States) for 2 h.

### Animal Study

120 Female C57BL/6J mice (6–8 weeks of age) were obtained from Southern Medical University Animal Centre (Guangzhou, China) and maintained in a specific pathogen-free environment. All experiments were performed according to the guidelines for experimental animals and approved by the Institutional Animal Care and Use Committee of the Institute of Biophysics, Chinese Academy of Sciences. The mice were intratracheally administered with either bleomycin (BLM, 3 mg/kg) or vehicle on Day 0. In the TUDCA prevention model, mice were first randomly assigned into four groups (*n* = 10 for each group): vehicle, TUDCA, BLM and BLM + TUDCA. TUDCA (50 mg/kg) was intraperitoneal injected at an interval of 1 day from Day1. Mice were sacrificed 3 weeks after TUDCA treatment. For the 1G6-D7 treatment model, 7 days after delivery of BLM, 3 weeks after 1G6-D7 nasal inhalation treatment, the mice were sacrificed and lungs were collected. The protocol of 1G6-D7 prevention model was reported previously (Dong et al., 2017). Lung microsections (5 μm) were stained with Masson’s trichrome and hematoxylin and eosin (H&E) to visualize fibrotic lesions.

### Cell Counting Kit-8 Assay

The cells were seeded in a 96-well plate, and then treated with different concentrations of rHSP90α to evaluate cell viability at different time points. Cell proliferation was detected by CCK8 (Dojindo, Japan) following the manufacturer’s protocol.

### EdU Assay

EdU assay was performed according to the manufacturer’s instructions of the EdU kit (Beyotime, China). The EdU reagent was diluted to 20 μM in serum-free medium, added to the cells and incubated for 4 h. After PBS washing, cells were fixed in 4% paraformaldehyde for 30 min and permeabilized with 0.3% Triton X-100 for 15 min. Dye these cells with Click Additive Solution according to the instructions. DAPI was added to stain the nucleus for 10 min. Finally, positive cells were counted by fluorescence microscope.

### Wound Healing Assay

IMR90 cells were seeded in six-well plates. When cells were grown to about 90% confluency and then scratched with a sterile 100 μl pipette tip. The cells were washed with PBS three times. Images of the wounded area were created at indicated time points with the same microscopic cross point by light microscopy.

### Immunofluorescence Staining

IMR90 cells were fixed in 4% paraformaldehyde for 30 min, permeabilized with 0.1% Triton X-100 for 20 min and then blocked with 1% BSA for 30 min. Cells were incubated with α-SMA and Collagen I were visualized with an overnight with specific fluorochrome primary antibodies including α-SMA (Abcam, United States), Collagen I (Affinity, China) at a concentration of 1:100. After extensive washing with PBS, cells were incubated with goat Alexa Fluor 488-labeled secondary antibody (Life Technologies, United States) for 1 h at room temperature and nuclei were stained with DAPI. The images were obtained by using Olympus FluoView^®^ FV1200 confocal laser scanning microscope (Olympus Corporation, Center Valley, PA).

### Western Blot Analysis

Lung tissues and cultured cells were extracted with RIPA buffer and then centrifuged at 15,000 rpm, 4°C for 15 min, the supernatant was collected. Protein concentration was quantified using a Bradford protein assay Kit (Beyotime Biotechnology, Shanghai, China). Equal amounts of protein were separated on SDS-PAGE, transferred onto PVDF membranes and then incubated with primary antibodies (Table 1). After being washed with TBST three times, membranes were then incubated with IRDye® 800CW- or 680RD- conjugated secondary antibodies and visualized using a LI-COR Odyssey Imaging System (LI-COR Biosciences, Lincoln, NE, United States).

**TABLE 1:** Antibody information.

**Antibody**	**CAS No**	**Company**
CollagenI	AF7001	Affinity
α-SMA	Ab5694	Abcam
GRP78	Sc-376768	Santa cruz
ATF6	Sc-1666659	Santa cruz
IRE1A	Sc-390960	Santa cruz
HSP90α	Ab59459	Abcam
β-actin	6008-1-Ig	Proteintech
AKT	4685s	CST
*p*-AKT (Ser473)	4060s	CST
*p*-AKT (Thr308)	2965s	CST
Alexa Fluor 488	A32723	ThermoFisher

### RNA-Seq

RNA was isolated from three biological replicates in both untreated and rHSP90α-treated group according to the manufacturer’s instructions. The cDNA fragments were purified and enriched by PCR to construct the cDNA library. Finally, the cDNA library was sequenced on the Illumina sequencing platform (Illumina HiSeq ™ 4000). The threshold of the *p*-value in multiple tests was determined by the false discovery rate (FDR). A threshold of the FDR ≤0.05 was used to judge the significance of gene expression differences. The RNA-seq data was uploaded to SRA database. Accession to cite for these SRA data: PRJNA716070.

### RNAi and Transfection

siRNAs were synthesized by GenePharma (Shanghai, China). The sequences used are show in Table 2. IMR90 cells transfections were conducted using Lipo3000 (Thermo Fisher Scientific) following the manufacturer’s protocol.

**TABLE 2:** The sequences of siRNA.

GRP78 siRNA-1	GAG​GCU​UAU​UUG​GGA​AAG​ATT (5^′^ to 3^′^)
	UCU​UUC​CCA​AAU​AAG​CCU​CTT (5^′^ to 3^′^)
GRP78 siRNA-2	GGG​CAA​AGA​UGU​CAG​GAA​ATT (5^′^ to 3^′^)
	UUU​CCU​GAC​AUC​UUU​GCC​CTT (5^′^ to 3^′^)
GRP78 siRNA-3	GAG​GUG​UCA​UGA​CCA​AAC​UTT (5^′^ to 3^′^)
	AGU​UUG​GUC​AUG​ACA​CCU​CTT (5^′^ to 3^′^)
Negative control	UUC​UCC​GAA​CGU​GUC​ACG​UTT (5^′^ to 3^′^)
	ACG​UGA​CAC​GUU​CGG​AGA​ATT (5^′^ to 3^′^)

### Immunohistochemistry

The expressions of α-SMA, GRP78 and HSP90α were characterized by immunohistochemistry using specific antibodies. Briefly, lung slices were dewaxed in xylene, followed by antigen retrieval with citrate buffer (pH 6.0) and incubated overnight with antibodies against α-SMA (Abcam, 1:400), GRP78 (Santa Cruz, 1:50) and HSP90α (Abcam, 1:200). Then, lung slices were incubated with secondary antibody for 30 min and visualized with a DAB substrate kit (Zhong Shan Jin Qiao, Beijing, China).

### ER-Tracker

ER-Tracker was performed to detect ER activity according to the instruction of the ER-Tracker kit (C1041, Beyotime, China). Briefly, cells were incubated with ER-Tracker working fluid for 20 min, followed by image acquisition.

### Quantitative RT-PCR

Lung fibroblasts were transfected with siRNA for 24 h and extracted the RNA with Trizol reagent (Takara, Japan). The SYBR Premix Ex Taq II Kit (Takara, Japna) was used to detect the expression of GRP78, normalized to the expression of the endogenous control GAPDH. The primer sequences were GRP78: 5′-ACC​TCC​AAC​CCC​GAG​AAC​A-3′ (forward), 5′-TTC​AAC​CAC​CTT​GAA​CGG​C-3′ (reverse); GAPDH:5′-AATTCCATGGCACCGTCAAG-3′ (forward), 5′-GGT​GAA​GAC​GCC​AGT​GGA​CT-3′ (reverse).

### Enzyme-Linked Immunosorbent Assay

Bronchoalveolar lavage fluid (BALF) and serum samples were collected as described previously (Yao et al., 2016). All the samples were centrifuged and the supernatant was collected and stored at −80°C until further analysis. The HSP90α (Cloud-Clone, Buckingham, United Kingdom) ELISA kit was used according to the manufacturer’s instruction.

### Preparation of Conditioned Media

The conditioned media was collected as previously described (Li et al., 2007) and then utilized to evaluate secretion of HSP90α.

### Statistical Analysis

All the experiments were conducted at least in triplicate. The data were presented as the means ± SEM or means ± SD. Data were analyzed with the use of an unpaired *t* test for comparisons between two conditions or ANOVA with the Tukey post test to determine the differences among all groups. The data of *in vivo* experiments were analyzed with the one-way ANOVA. The significance level was set at *p* < 0.05. Statistical analysis was performed using GraphPad Prism software (GraphPad Software, United States).

## Results

### Extracellular HSP90α Promotes Lung Fibroblasts Activation But Have No Influence on Proliferation

**FIGURE 1:**
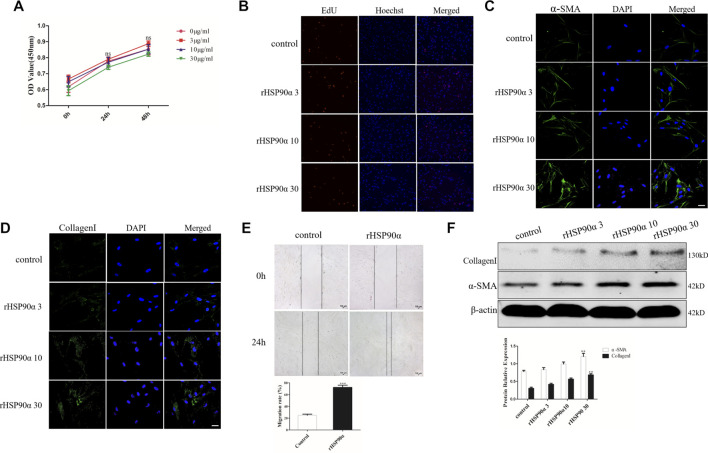
Extracellular HSP90α promotes lung fibroblasts activation but has no influence on proliferation. **(A)**. Cell proliferation was assessed *via* the CCK8 assay after stimulation with 0, 3, 10 and 30 ug/ml rHSP90α for indicated times (24 and 48 h) in the IMR90 cells. **(B)**. Proliferative capacity was analyzed using an EdU assay after treatment with 0, 3, 10 and 30 ug/ml rHSP90α in the IMR90 cells for 24 h. Lung fibroblasts activation was assessed by immunofluorescence staining for α-SMA and CollagenI **(C,D)**, representative staining images of α-SMA–positive stress fibers (green), CollagenI-positive collagen deposition (green) and DAPI (blue) showing nuclei under confocal laser scanning microscopy (scale bar = 50 μm). **(E)**. Fibroblasts migration were performed by wound-healing assays. **(F)**. The protein expression of CollagenI and α-SMA were determined by western blot after stimulation of different concentrations of rHSP90α for 24 h, β-actin was used as an internal control. **ns** = no significance, *p < 0.05, **p < 0.01.

Pulmonary fibrosis is characterized by the proliferation and differentiation of lung fibroblasts (Penke et al., 2018). To evaluate the role of eHSP90α in the pulmonary fibrosis, the effect of eHSP90α on fibroblasts proliferation and differentiation was measured first. Lung fibroblasts were treated with different concentrations of eHSP90α for the indicated times. Proliferation ability was determined by the CCK8 assay. As shown in Figure 1A, there was no significant difference between the rHSP90α-treated and untreated groups. In addition, the EdU assay was performed, and the EdU-positive cells in the rHSP90α-treated groups showed no obvious differences in comparison with the control group (Figure 1B). The differentiation of fibroblasts to myofibroblasts is accompanied by an increase in α-SMA and collagen I expression and migration (Chen et al., 2019). Next, to test the expression of eHSP90α on myofibroblast markers, lung fibroblasts were treated with different concentrations of rHSP90α for 24 h and evaluated by immunofluorescence staining. The results showed that α-SMA and collagen I expression increased in a concentration-dependent manner in comparison with the control group (Figures 1C,D). Next, to investigate whether eHSP90α affects lung fibroblast migration, a wound-healing assay was performed. As shown in Figure 1E, rHSP90α significantly promoted the migration of lung fibroblasts. Consistently, western blotting analysis confirmed that the expression of α-SMA and collagen I increased with increasing concentrations of eHSP90α (Figure 1F). Taken together, these results showed that eHSP90α could activate lung fibroblasts but had no obvious influence on proliferation.

### Extracellular HSP90α Induces ER Stress in Lung Fibroblasts

**FIGURE 2:**
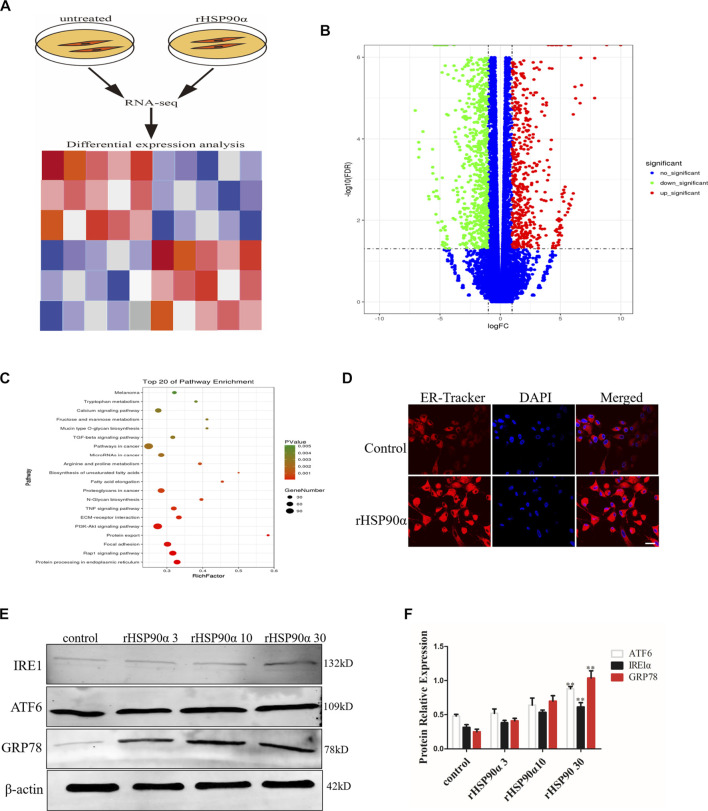
Extracellular HSP90α induces ER Stress in lung fibroblasts. **(A)**. Schematic diagram of RNA-seq with or without rHSP90α treatment. **(B)**. Volcano plot displays the overall genes identified with a *p* < 0.05 and |log2FC|>1.0 a cutoff. **(C)**. KEGG pathway analysis of pathway enrichment. The vertical axis represents the pathway category and the horizontal axis represents the enrichment score [−log (*p*-value)] of the pathway. Significantly enriched KEGG pathways (*p* < 0.05) are presented. The data were analyzed by DAVID bioinformatics tools. **(D)**. Endoplasmic reticulum (ER) activity was assessed by immunofluorescence staining ER-Tracker. Representative staining images of ER-positive cells and DAPI (blue) showing nuclei under confocal laser scanning microscopy (scale bar = 50 μm). **(E,F)**. Western blot analysis of expression of ATF6, IRE1α and GRP78 after different concentrations of rHSP90α treatment for 24 h, β-actin was used as an internal control. *p < 0.05, **p < 0.01.

To further explore the potential mechanisms by which eHSP90α promotes fibroblast activation, RNA-seq was performed in lung fibroblasts with or without rHSP90α treatment (Figure 2A). According to the cut-off criteria of *p* < 0.05 and |log2FC|>1.0, 4905 dysregulated genes were identified (Figure 2B). KEGG pathway enrichment analysis showed that these genes were principally categorized into regulation of protein processing in the ER, focal adhesion, and PI3K-AKT pathway. To validate ER activity in rHSP90α-treated fibroblasts, ER-Tracker staining was performed. As shown in Figure 2D, treatment of lung fibroblasts with rHSP90α for 24 h significantly increased the ER-Tracker staining intensity. In addition, we stimulated lung fibroblasts with different concentrations of rHSP90α for 24 h and found that the ER stress markers GRP78, ATF6, IRE1α upregulated effectively (Figures 2E,F). These data suggested that eHSP90α could induce the ER stress in the lung fibroblasts.

### ER Stress Mediated Lung Fibroblasts Activation in Pulmonary Fibrosis

**FIGURE 3:**
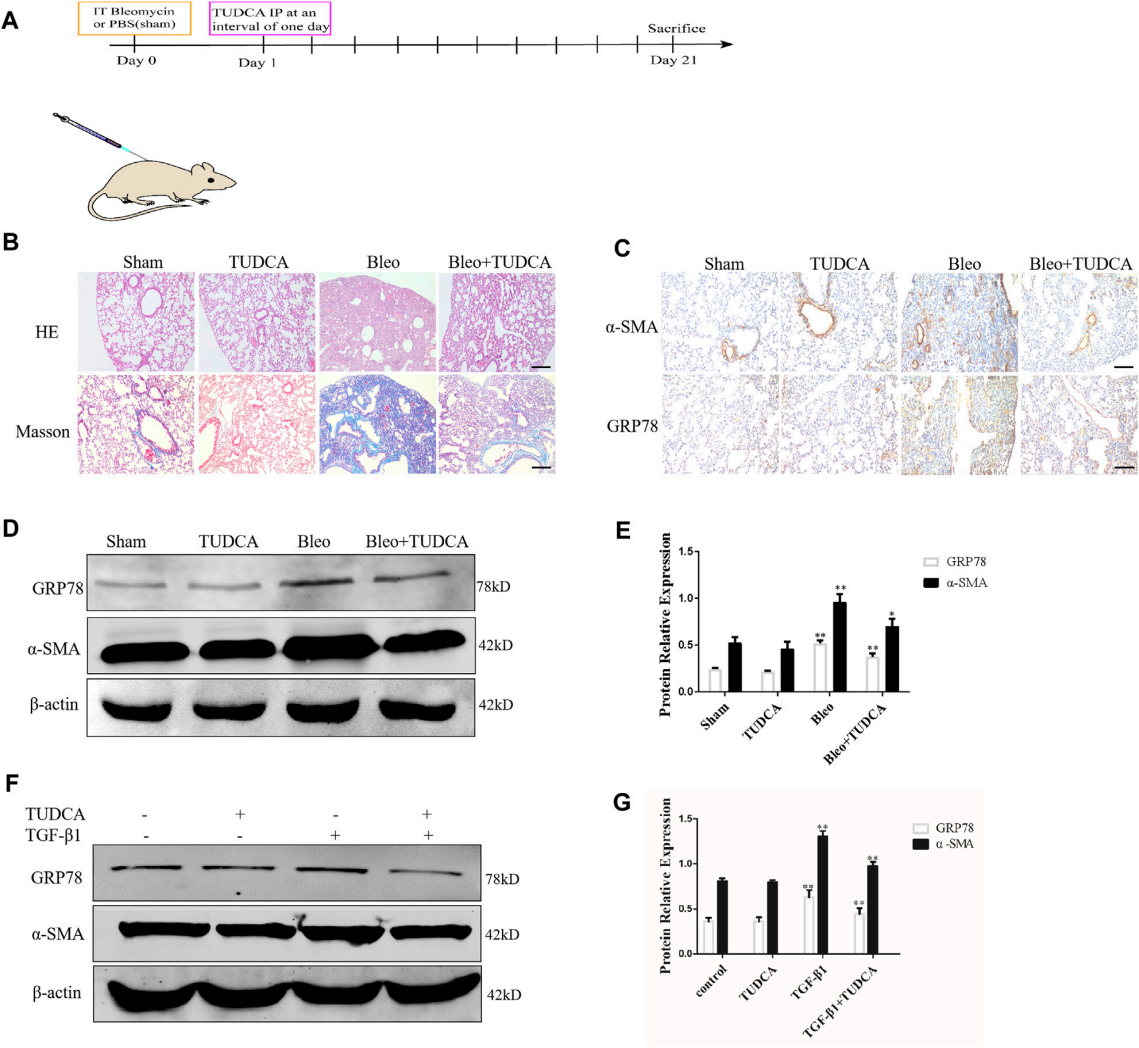
ER stress in the lung fibroblasts is critical for pulmonary fibrosis progression. **(A)** Experimental scheme of the mouse model of bleomycin-induced pulmonary fibrosis. Mice were intratracheally injected with saline or bleomycin (3 mg/kg) at day 0. On day 1, mice were administrated with TUDCA (50 mg/kg) or DMSO by intraperitoneal injection every 2 days. Mice were sacrificed on day 21 (*n* = 10 for each group). **(B)** Histological images and collagen deposition of the lung tissue was detected by H&E and Masson staining. Scale bar = 100 μm. **(C)** Representative images showing GRP78 and α-SMA staining of lung tissues of mice treated with saline, bleomycin without or with TUDCA. Scale bar = 100 μm. **(D, E)** Western blot analysis of expression of GRP78 and α -SMA. **(F, G)**, IMR90 were pre-treated with TUDCA (100 μM) for 2 h and followed by TGF-β1 (10 ng/mL) for 24 h. The expression levels of GRP78 and α-SMA was measured by Western blot. β-actin was used as an internal control. *p < 0.05, **p < 0.01.

To determine whether ER stress is involved in lung fibroblast activation in pulmonary fibrosis, we established a mouse model of lung fibrosis induced by intratracheal instillation of bleomycin. TUDCA, an ER stress inhibitor, was intraperitoneally injected at 1 d intervals from Day 1 (Figure 3A). As expected, H&E and Masson staining revealed that TUDCA effectively ameliorated the distorted alveolar structure, thickened alveolar walls and collagen deposition induced by BLM (Figure 3B). In addition, IHC staining results showed that TUDCA significantly decreased the GRP78 expression, particularly in the α-SMA positive fibrotic foci (Figure 3C). Similarly, western blotting results showed that TUDCA downregulated BLM-stimulated α-SMA and GRP78 expression (Figures 3D,E). We used TGF-β1 to treat human lung fibroblasts as an *in vitro* model. As shown in Figures 3F,G, TGF-β1 treatment in lung fibroblasts increased the expression of the ER stress marker GRP78 and myofibroblast marker α-SMA, whereas the expression of these markers was attenuated by TUDCA treatment (100 μM). Taken together, these data suggest that ER stress plays a crucial role in lung fibroblast activation.

### Extracellular HSP90α Activates Lung Fibroblasts *via* ER Stress

**FIGURE 4:**
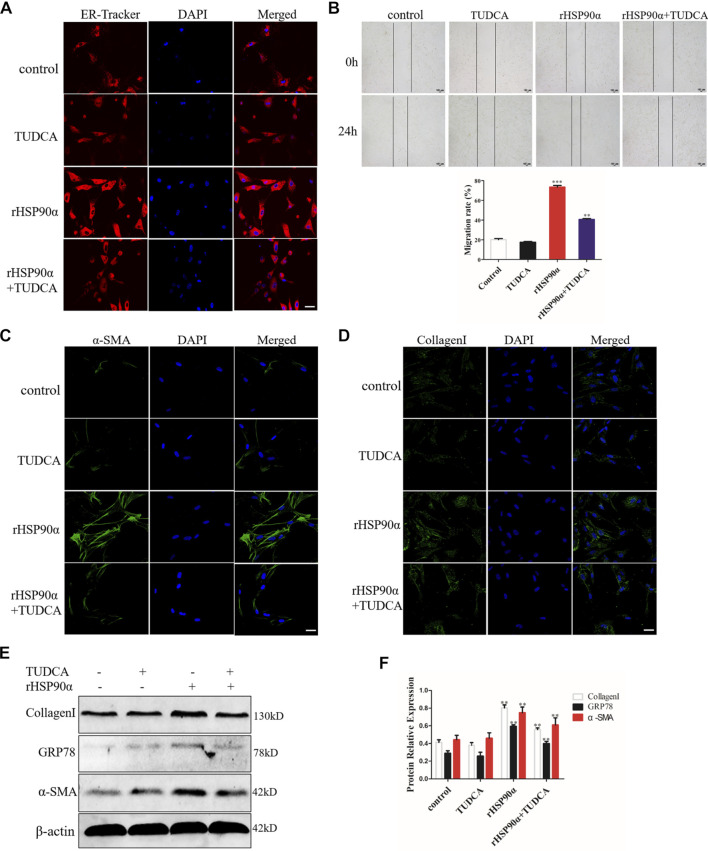
Extracellular HSP90α activates lung fibroblasts *via* ER Stress. **(A)**. Representative images showing ER-Tracker staining of IMR90 cells pre-treated with or without TUDCA (100 μM) and followed by rHSP90α for 24 h, scale bar = 50 μm. **(B)**. Cell migration was examined in IMR90 cells by a wound healing assay. **(C,D)**. Representative images showing immunofluorescence staining of α-SMA and CollagenI in IMR90 cells. **(E,F)**. Western blot analysis of the expression of CollagenI, GRP78 and α-SMA. β-actin was used as an internal control. *p < 0.05, **p < 0.01.

The above data showed that the most significant pathway enrichment between the untreated group and the rHSP90α-treated group was protein processing in the ER. Therefore, we speculated that eHSP90α activated fibroblasts and promoted fibrosis by inducing ER stress. To test this assumption, we first used ER-Tracker to detect the ER activity. As shown in Figure 4A, we found that TUDCA effectively abrogated the staining intensity of ER-Tracker, which was increased by eHSP90α. In addition, wound healing results showed that lung fibroblast migration was markedly increased by eHSP90α stimulation, while TUDCA alleviated this effect (Figure 4B). We further used immunofluorescence staining to examine α-SMA and collagen I expression and observed lower α-SMA positive cells and less collagen deposition in the rHSP90α+TUDCA group than in the rHSP90α group (Figures 4C,D). Consistent with these observations, western blotting analysis indicated that TUDCA significantly reduced rHSP90α-induced α-SMA and collagen І expression (Figures 4E,F). Taken together, these results suggest that eHSP90α promotes lung fibroblast differentiation by activating ER stress.

### Knockdown of GRP78 Abrogates Lung Fibroblast Activation Induced by eHSP90α

**FIGURE 5:**
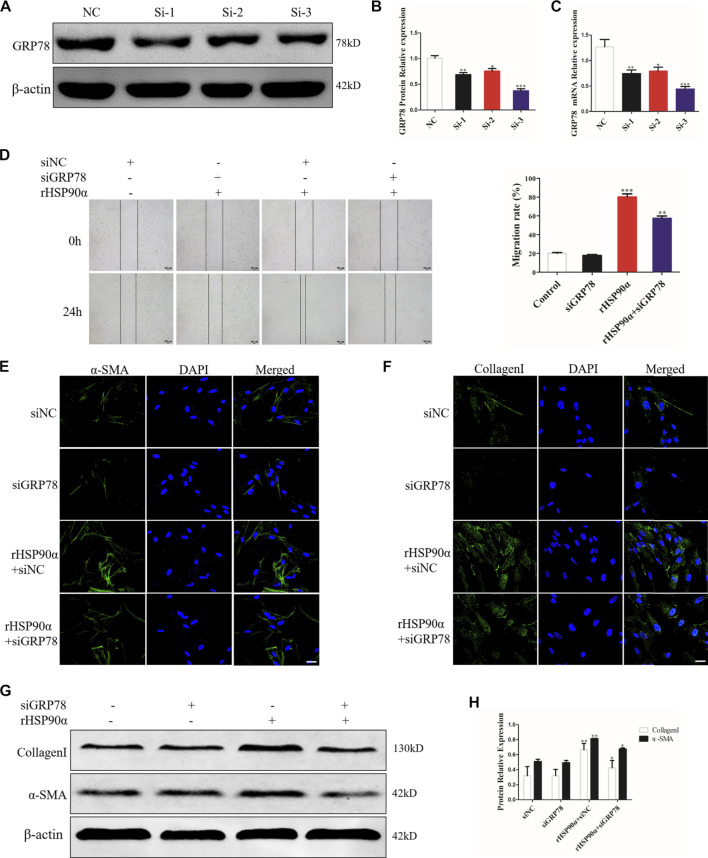
GRP78 is essential for lung fibroblast activation induced by eHSP90α. IMR90 cells were transfected with siGRP78 or siNC and the efficiency was assessed by western blot **(A,B)** and qPCR **(C)**. Cell migration was examined in IMR90 cells by a wound healing assay **(D)**. Lung fibroblasts activation was assessed by immunofluorescence staining for α-SMA and CollagenI **(E,F)**, representative staining images of α-SMA–positive stress fibers (green), CollagenI-positive collagen deposition (green) and DAPI (blue) showing nuclei under confocal laser scanning microscopy (scale bar = 50 μm). **(G,H)**. The expression of CollagenI and α-SMA were measured by western blot. β-actin was used as an internal control. *p < 0.05, **p < 0.01.

GRP78 is a crucial modulator of the ER that responds to UPR and maintains cellular homeostasis, contributing to proliferation and differentiation (Aran et al., 2018; van Lidth et al., 2018; Du T et al., 2019; Merkel et al., 2019). Thus, we hypothesized that eHSP90α induces ER stress to further activate fibroblasts by upregulating GRP78 expression. To confirm our assumption, we designed three siRNAs and transfected them into lung fibroblasts to knock down GRP78. The interference efficiency was verified using western blotting and qRT-PCR. As shown in Figure 5A,C, the results revealed that the relative level of GRP78 was significantly decreased by the siRNAs. Thus, si-3 was selected as the target siRNA for GRP78. Next, GRP78 was knocked down in lung fibroblasts with siRNA, followed by rHSP90α stimulation. As shown in Figure 5D, GRP78 depletion markedly abrogated the effects of eHSP90α on cell migration. In addition, knockdown GRP78 significantly reduced α-SMA staining intensity and collagen deposition induced by eHSP90α in fibroblasts (Figures 5E,F). Consistent with the immunofluorescence staining results, the protein expression of α-SMA and collagen I upregulated by eHSP90α was effectively attenuated by depletion of GRP78 (Figures 5G,H). These data strongly suggest that GRP78 is essential for eHSP90α-induced lung fibroblast activation and ECM production.

### The Monoclonal Antibody 1G6-D7 Attenuates Pulmonary Fibrosis by Decreasing ER Stress *in vitro* and *in vivo*

**FIGURE 6:**
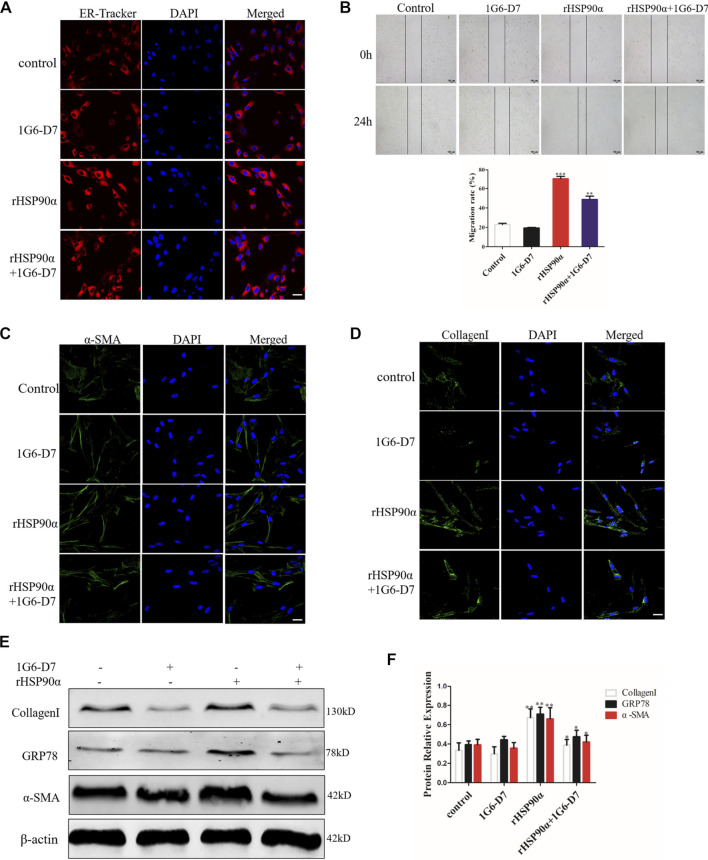
Monoclonal antibody 1G6-D7 inhibits ER stress induced by eHSP90α ***in vitro*. (A)**. Representative images showing ER-Tracker staining of IMR90 cells pre-treated with or without 1G6-D7 (10 μg/ml) and followed by rHSP90α for 24 h, scale bar = 50 μm. **(B)** Cell migration was examined in IMR90 cells by a wound healing assay. **(C,D)**. Representative images showing immunofluorescence staining of α -SMA and CollagenI in IMR90 cells. **(E,F)**. Western blot analysis of the expression of CollagenI, GRP78 and α-SMA. β-actin was used as an internal control. *p < 0.05, **p < 0.01.

**FIGURE 7:**
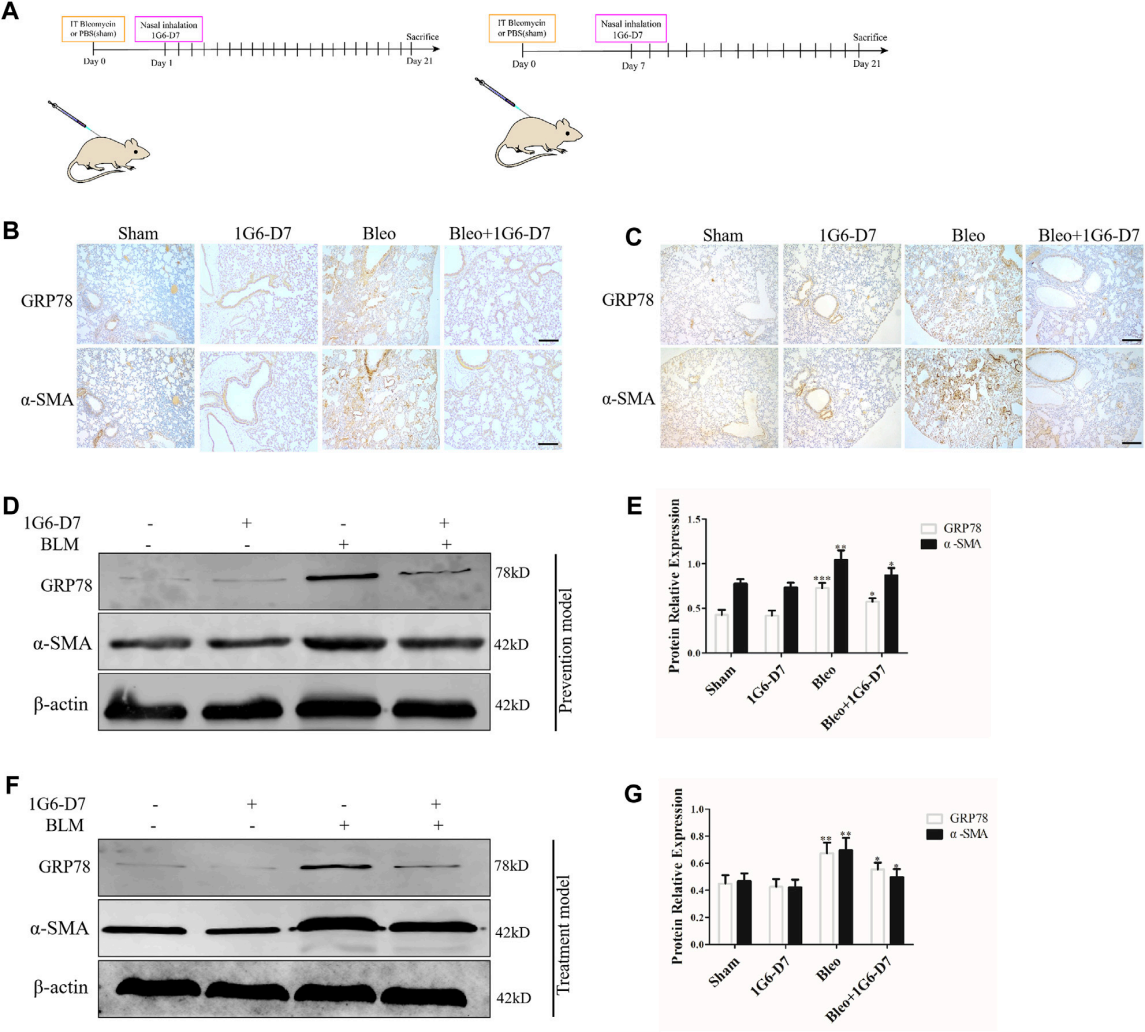
Monoclonal antibody 1G6-D7 inhibits ER stress in the bleomycin-induced pulmonary fibrosis model. **(A)** Schematic diagram of mouse model establishment (n = 10 for each group). **(B)** Representative images showing GRP78 and α-SMA staining of lung tissues of mice in the prophylactical model. Scale bar = 100 μm. **(C)** Representative images showing GRP78 and α-SMA staining of lung tissues of mice in the therapeutical model. Scale bar = 100 μm. Western blot analysis of the expression of GRP78 and α-SMA in the prophylactical model **(D, E)** and therapeutical model **(F, G)**. β-actin was used as an internal control. *p < 0.05, **p < 0.01.

1G6-D7, a selective anti-HSP90α monoclonal antibody, was previously reported to attenuate pulmonary fibrosis by inhibiting the MAPK signaling pathway (Dong et al., 2017). However, whether 1G6-D7 abrogated the ER stress and fibroblast activation induced by eHSP90α remains unclear. First, lung fibroblasts were pre-treated with 1G6-D7 and followed by rHSP90α for 24h, and ER-Tracker staining was used to examine the role of 1G6-D7 on ER activity. As shown in Figure 6A, 1G6-D7 significantly decreased the staining intensity induced by rHSP90α. Next, a wound-healing assay was performed to detect the effect of 1G6-D7 on the migration of lung fibroblasts. As shown in Figure 6B, 1G6-D7 remarkably inhibited the migration stimulated by rHSP90α. We further found that treatment with 1G6-D7 inhibited the effects of fibroblast activation by preventing α-SMA and collagen upregulation (Figures 6C,D). Consistently, western blot results showed that 1G6-D7 effectively downregulated the expression of GRP78, collagen I and α-SMA induced by rHSP90α. *In vivo*, we established prophylactical and therapeutical models to confirm the effect of 1G6-D7 on BLM-induced pulmonary fibrosis (Figure 7A). As shown in Figure 7B, IHC was performed to examine GRP78 and α-SMA in the cortical model. We observed that 1G6-D7 significantly decreased the GRP78 expression, particularly in the α-SMA positive fibrotic foci. In the therapeutical model, blocking HSP90α with 1G6-D7 similarly decreased the GRP78 and α-SMA expression through IHC (Figure 7C). Western blotting results showed that 1G6-D7 downregulated the expression of GRP78 and α-SMA induced by BLM in the prevention model (Figures 7D,E). Consistently, we found that 1G6-D7 also significantly inhibited the upregulation of GRP78 and α-SMA upon the BLM treatment in the treatment model (Figures 7F,G). These results demonstrates that 1G6-D7 attenuates the pulmonary fibrosis by inhibiting ER stress and that 1G6-D7 might be a potential therapeutic agent for pulmonary fibrosis patients.

### Extracellular HSP90α Facilitates ER Stress Through the PI3K/AKT Pathway

**FIGURE 8:**
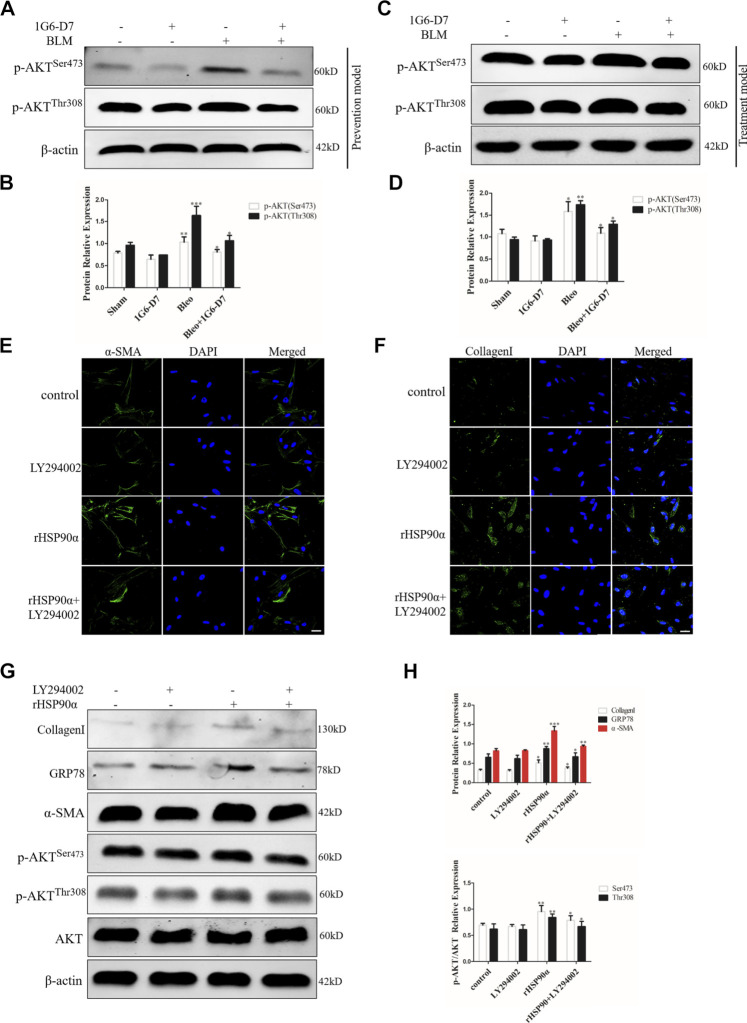
PI3K/AKT signaling pathway is involved in ER stress induced by eHSP90α. The expression of *p*-AKT^Ser473^ and *p*-AKT^Thr308^ was measured by western blot in the prophylactical model **(A,B)** and in the therapeutical model **(C,D)**. Immunofluorescence staining showed that α -SMA and CollagenI in IMR90 cells pre-treated with or without LY294002 (10 μM) followed by rHSP90α stimuli **(E,F)**. Scale bar = 50 μm. **(G,H)**. Western blot analysis of the expression of CollagenI, GRP78, α-SMA, *p*-AKT^Ser473^, *p*-AKT^Thr308^ and AKT. β-actin was used as an internal control. *p < 0.05, **p < 0.01, ***p < 0.001.

Several studies have revealed that the PI3K/AKT signaling pathway is involved in regulating ER stress (Hsu et al., 2017). However, whether eHSP90α induces ER stress through PI3K/AKT signaling pathway has not been clarified. Based on the KEGG pathway enrichment analysis, PI3K/AKT signaling pathway was found to be significantly enriched among differentially expressed genes between the rHSP90α-treated group and the untreated group. We first examined the phosphorylation of AKT *in vivo* by using western blot. As shown in Figures 8A,B, phosphorylation of AKT were upregulated by BLM, but was significantly attenuated by the monoclonal antibody 1G6-D7 in the prevention model. In the treatment model, 1G6-D7 effectively reduced the phosphorylation of AKT (Figures 8C,D). In addition, immunofluorescence staining results showed that the PI3K/AKT inhibitor (LY294002) largely abolished the effect of rHSP90α on increasing the α-SMA and collagen I expression (Figures 8E,F). We further examined the effect of LY294002 on ER stress and fibroblast activation induced by rHSP90α. As shown in Figures 8G,H, pre-treatment with LY294002 effectively reduced the ER stress marker GRP78 and the increased phosphorylation of Akt induced by rHSP90α. Western blotting analysis also showed that pre-treatment with LY294002 significantly downregulated the expression of α-SMA and collagen I following treatment with rHSP90α. Collectively, these data suggest that eHSP90α induces ER stress, promotes fibroblast activation *via* the PI3K/AKT pathway, and inhibited PI3K/AKT, with LY294002 significantly attenuates the ER stress and fibroblasts activation induced by eHSP90α.

### ER Stress Inhibitor TUDCA Suppress Extracellular HSP90α Secretion

**FIGURE 9:**
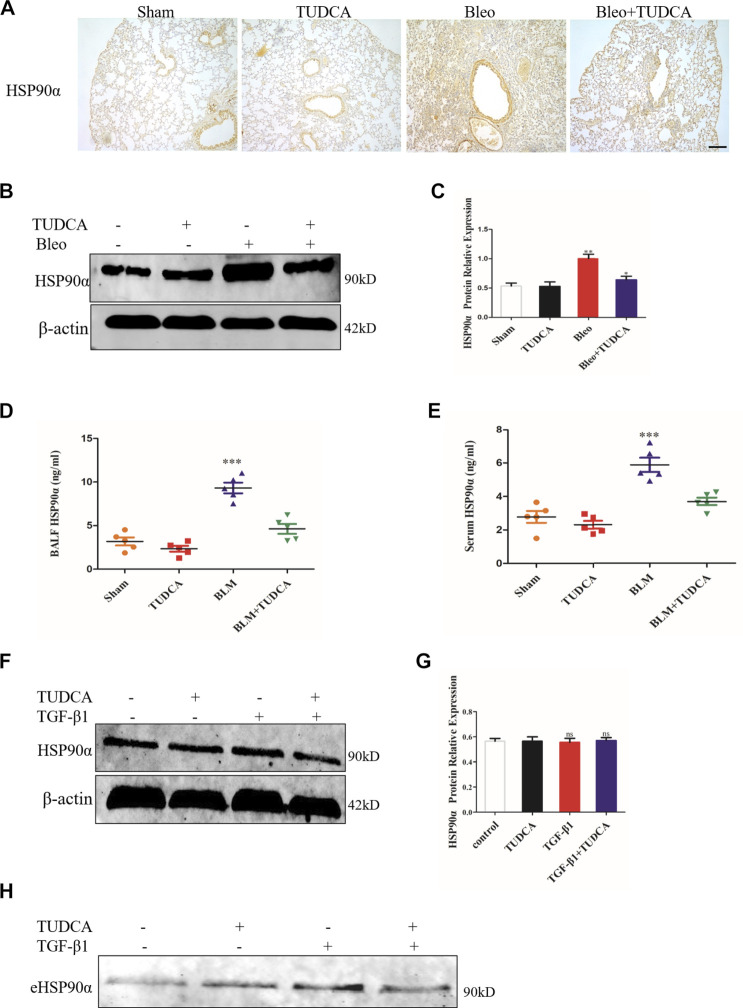
TUDCA significantly decreased the extracellular HSP90α secretion. **(A)**. Representative images showing HSP90α staining of lung tissues of mice. Scale bar = 100 μm. **(B,C)**. The expression of HSP90α was assessed by western blot. The content of HSP90α in BALF **(D)** and in serum **(E)** samples in the mice were measured by ELISA (*n* = 5 for each group). **(F,G)**. Western blot analysis of the expression of HSP90α in IMR90 cells pre-treated with TUDCA (100 μM) followed by TGF-β1 stimuli. β-actin was used as an internal control. **(H)**. Secretion of HSP90α in IMR90 cells pre-treated with TUDCA followed by TGF-β1 treatment was detected by western blot. **ns** = no significance, *p < 0.05, **p < 0.01, ***p < 0.001.

Some studies have reported that ER stress could regulate cellular homeostasis and stimulate extracellular vesicle secretion, and eHSP90α was also reported to be secreted through exosomes (Kakazu et al., 2016a; Guo et al., 2017; Zhang et al., 2017; Liu et al., 2019). In addition, previous studies demonstrated that TGF-β1 or BLM increased the secretion of eHSP90α in a pulmonary fibrosis model (Dong et al., 2017). Therefore, we hypothesized that eHSP90α secretion may respond to ER stress in pulmonary fibrosis. We first detected the expression of HSP90α by using IHC. As shown in Figure 9A, BLM significantly increased the expression of HSP90α and was abrogated by TUDCA. Similarly, western blotting results showed that TUDCA markedly decreased the BLM-induced expression of HSP90α (Figures 9B,C). Furthermore, eHSP90α levels were examined using ELISA, and TUDCA was found to effectively decrease BLM-induced eHSP90α content in both BALF and serum (Figures 9D,E). Moreover, to elucidate whether TUDCA can inhibit eHSP90α secretion *in vitro*, we pre-treated the lung fibroblasts with TUDCA, followed by TGF-β1. As shown in Figures 9F,G, cellular HSP90α expression was not significantly different between the TGF-β1 and the TUDCA + TGF-β1 groups. However, we were surprised to find that TUDCA remarkably inhibited the secretion of eHSP90α (Figure 9H). These results suggest that eHSP90α secretion is associated with ER stress, and that inhibition of ER stress by TUDCA can effectively reduce eHSP90α in the pulmonary fibrosis.

## Discussion

**FIGURE 10:**
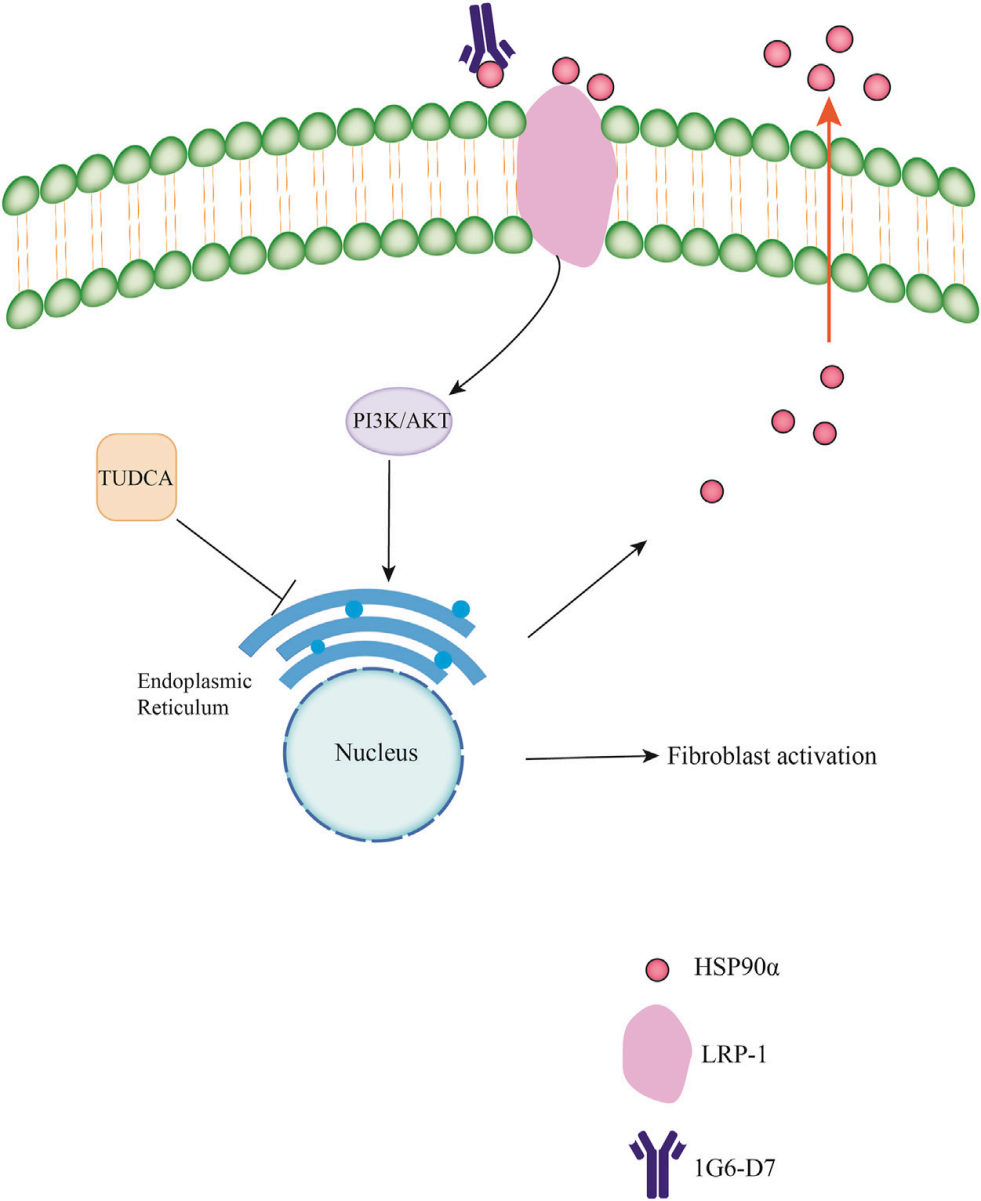
Schematic diagram of the molecular mechanisms underlying the extracellular HSP90αinteracts with ER stress to promote fibroblasts activation through PI3K/AKT pathway in pulmonary fibrosis.

Pulmonary fibrosis is mainly characterized by alveolar injury, fibroblast activation, proliferation, and ECM accumulation. Fibroblasts/myofibroblasts play an essential role in the progression of pulmonary fibrosis. As a member of the heat shock protein family, the role of HSP90α in cancer progression, fibrosis, and diabetes has been widely investigated (Cheng et al., 2011; Bonniaud et al., 2018; Zhou et al., 2019). The main function of HSP90α is to regulate cell proliferation, differentiation, and epithelial mesenchymal transition (As et al., 2004). Notably, HSP90α can be secreted into the extracellular space to exert its function by interacting with LDL Receptor–Related Protein 1 (LRP-1) (Chen et al., 2010). We previously reported that eHSP90α promotes pulmonary fibrosis by activating the MAPK signaling pathway (Dong et al., 2017). In addition, Bellaye et al. also found that eHSP90α was strongly associated with disease severity in pulmonary fibrosis and promoted pulmonary fibrosis *via* LRP-1 (Bellaye et al., 2018). Thus, eHSP90α may play a crucial role in pulmonary fibrosis. Our study aimed to explore the molecular mechanisms underlying the effects of eHSP90α in pulmonary fibrosis. In this study, we demonstrated that eHSP90α promoted fibroblast activation by inducing ER stress *via* the PI3K/AKT signaling pathway. We also examined the relationship between eHSP90α secretion and ER stress and observed that eHSP90α secretion could be regulated by ER stress (Figure 10).

ER stress can be induced by several pathological stimuli, including glucose starvation, hypoxia and oxidative stress (Yoshida, 2007; Cao and Kaufman, 2014). Emerging evidence has demonstrated that ER stress can regulate cell differentiation, including the differentiation of lung fibroblasts (Matsuzaki et al., 2015; Tanimura et al., 2018; Peñaranda-Fajardo et al., 2019). However, whether ER stress is involved in the effect of eHSP90α on pulmonary fibrosis has not been fully clarified. In this study, we found that the expression of ER stress-related proteins GRP78, IRE1α and ATF6 was significantly higher in rHSP90α-treated IMR90 cells compared to untreated IMR90 cells. GRP78 is a key modulator that assists in the correct folding of newly synthesized proteins. Our results showed that GRP78 was upregulated in activated fibroblasts both *in vitro* or *in vivo*. Depletion of GRP78 strikingly inhibited eHSP90α-induced fibroblast differentiation and ECM deposition. Consistently, a recent study confirmed that cigarette smoke extract could promote human lung myofibroblast differentiation through GRP78 upregulation (Song et al., 2019a).Interestingly, GRP78 was contradictorily downregulated in the type II alveolar epithelial cells of patients with IPF (Borok et al., 2020). By combining these two results, we speculated that GRP78 might play distinct roles in different cells, and we would attempt to explore its mechanism. Furthermore, inhibiting ER stress with TUDCA remarkedly attenuated fibroblast activation and pulmonary fibrosis progression *in vitro* and *in vivo*. These findings elucidated the mechanism by which eHSP90α contributes to the development of pulmonary fibrosis by inducing ER stress in lung fibroblasts.

HSP90 inhibitors have been reported to be potential treatments for multiple cancers and pulmonary fibrosis (Trepel et al., 2010; Colunga et al., 2020). However, almost all the clinical trials have failed because of the pan-inhibitory activity of HSP90 inhibitors (Sanchez et al., 2020). Therefore, a selectively HSP90α-inhibiting agent is more suitable for pulmonary fibrosis treatment. We previously utilized monoclonal antibody 1G6-D7 to antagonize HSP90α to evaluate the effect of eHSP90α on pulmonary fibrosis in a prophylactical model. Although we observed that 1G6-D7 could protect against BLM-induced pulmonary fibrosis, whether 1G6-D7 played a similar role in the therapeutical model was not fully understood. In this study, we confirmed that administration of 1G6-D7 from Day7 to Day 21 after intratracheal BLM injection also effectively attenuated pulmonary fibrosis. We further demonstrated that 1G6-D7 decreased the expression of ER stress marker GRP78 in our model both prophylactically and therapeutically. Consistently, the effect of extracellular HSP90α on lung fibroblasts could be hampered by 1G6-D7 *in vitro*. Our findings suggest that antagonism with 1G6-D7 might have a potential antifibrotic effect on pulmonary fibrosis through inhibiting ER stress.

The PI3K/AKT pathway is the most commonly signaling pathway in pulmonary diseases, including pulmonary fibrosis (Hsu et al., 2017; Wang et al., 2018; Shi et al., 2019; Wan et al., 2019). Several studies have suggested that the PI3K/AKT signaling pathway is particularly important in mediating ER stress in various diseases (Hsu et al., 2017; Song et al., 2019b; Wang et al., 2020). However, the mechanisms of the PI3K/AKT signaling pathway underlying the effect of eHSP90α on pulmonary fibrosis remain poorly understood. Our RNA-seq data showed that treatment of lung fibroblasts with rHSP90α activated the PI3K/AKT signaling pathway in comparison with the untreated group. Notably, by using a selective PI3K/AKT inhibitor (LY294002), we verified that the PI3K/AKT signaling pathway is essential for eHSP90α-induced fibroblast activation and ER stress. Similarly, a recent study indicated that ultrafine silicon dioxide nanoparticle could cause lung epithelial cells ER stress *via* the PI3K/AKT signaling pathway. Treatment with the ROS inhibitor N-acetyl-l-cysteine (NAC) and LY294002 reversed the signals induced by ultrafine silicon dioxide nanoparticle (Lee et al., 2020). Collectively, these findings suggest that eHSP90α activates ER stress and fibroblasts *via* the PI3K/AKT signaling pathway.

Some studies have demonstrated that ER stress could stimulate extracellular vesicle secretion to further promote cancer immune escape and inflammation (Dasgupta et al., 2020; Yao et al., 2020). We previously observed that eHSP90α secretion was increased whether in the lung fibroblasts stimulated by TGF-β1 or BALF/serum induced by BLM (Dong et al., 2017). TGF-β1 has been to induce ER stress in lung fibroblasts (Hsu et al., 2017). Thus, we speculated that the eHSP90α production might be regulated by ER stress. In our study, we discovered that treatment with TUDCA significantly decreased HSP90α levels in the BALF and serum. Intriguingly, TUDCA did not alter HSP90α expression at the intracellular level, but markedly reduced the eHSP90α content. Thus, these findings suggest that eHSP90α production is involved in ER stress in the pulmonary fibrosis.

However, one of the limitations of this study is that we were unable to demonstrate that the direct molecular mechanism by which ER stress regulates the eHSP90α secretion in pulmonary fibrosis. Several studies reported that exosome induced by ER stress was highly associated with IRE1α (Kakazu et al., 2016b; Hosoi et al., 2018; Xu et al., 2019). Future research will focus on whether eHSP90α secretion is IRE1α-dependent. This future direction may be important to better understand how eHSP90α regulates pulmonary fibrosis.

In summary, the present study demonstrated that eHSP90α promoted lung fibroblast activation in the pulmonary fibrosis by inducing ER stress *in vitro* and *in vivo*. The role of eHSP90α in ER stress is, at least partially, mediated by activation of the PI3K/Akt signaling pathway. The production of eHSP90α in the pulmonary fibrosis is mediated by ER stress activation. These observations strengthen our notion that eHSP90α interacts with ER stress to promote lung fibroblast activation in pulmonary fibrosis and provide a potential therapeutic strategy for pulmonary fibrosis.

## Data Availability

The datasets presented in this study can be found in online repositories. The names of the repository/repositories and accession number(s) can be found in the article/Supplementary material.

## References

[B1] Aran G. Sanjurjo L. Bárcena C. Simon-Coma M. Téllez É. Vázquez-Vitali M. (2018). CD5L Is Upregulated in Hepatocellular Carcinoma and Promotes Liver Cancer Cell Proliferation and Antiapoptotic Responses by Binding to HSPA5 (GRP78). FASEB J. 32 (7), 3878–3891. 10.1096/fj.201700941RR 29465313

[B2] As S. Kalmár E. Csermely P. Yf S. (2004). Hsp90 Isoforms: Functions, Expression and Clinical Importance. FEBS Lett. 562, 11–15. 10.1016/s0014-5793(04)00229-710.1002/feb2.2004.562.issue-1-3 15069952

[B3] Bellaye P. S. Shimbori C. Yanagihara T. Carlson D. A. Hughes P. Upagupta C. (2018). Synergistic Role of HSP90α and HSP90β to Promote Myofibroblast Persistence in Lung Fibrosis. Eur. Respir. J. 51 (2). 10.1183/13993003.00386-2017 29386344

[B4] Bhakta N. R. Christenson S. A. Nerella S. Solberg O. D. Nguyen C. P. Choy D. F. (2018). IFN-stimulated Gene Expression, Type 2 Inflammation, and Endoplasmic Reticulum Stress in Asthma. Am. J. Respir. Crit. Care Med. 197 (3), 313–324. 10.1164/rccm.201706-1070OC 29064281 PMC5811952

[B5] Bonniaud P. Burgy O. Garrido C. (2018). Heat Shock Protein-90 toward Theranostics: a Breath of Fresh Air in Idiopathic Pulmonary Fibrosis. Eur. Respir. J. 51 (2). 10.1183/13993003.02612-2017 29437951

[B6] Borok Z. Horie M. Flodby P. Wang H. Liu Y. Ganesh S. (2020). Grp78 Loss in Epithelial Progenitors Reveals an Age-Linked Role for Endoplasmic Reticulum Stress in Pulmonary Fibrosis. Am. J. Respir. Crit. Care Med. 201 (2), 198–211. 10.1164/rccm.201902-0451OC 31738079 PMC6961744

[B7] Cao S. S. Kaufman R. J. (2014). Endoplasmic Reticulum Stress and Oxidative Stress in Cell Fate Decision and Human Disease. Antioxid. Redox Signal. 21 (3), 396–413. 10.1089/ars.2014.5851 24702237 PMC4076992

[B8] Carrasco D. R. Sukhdeo K. Protopopova M. Sinha R. Enos M. Carrasco D. E. (2007). The Differentiation and Stress Response Factor XBP-1 Drives Multiple Myeloma Pathogenesis. Cancer Cell 11 (4), 349–360. 10.1016/j.ccr.2007.02.015 17418411 PMC1885943

[B9] Chen J. S. Hsu Y. M. Chen C. C. Chen L. L. Lee C. C. Huang T. S. (2010). Secreted Heat Shock Protein 90alpha Induces Colorectal Cancer Cell Invasion through CD91/LRP-1 and NF-kappaB-Mediated Integrin alphaV Expression. J. Biol. Chem. 285 (33), 25458–25466. 10.1074/jbc.M110.139345 20558745 PMC2919109

[B10] Chen Y. Zhao X. Sun J. Su W. Zhang L. Li Y. (2019). YAP1/Twist Promotes Fibroblast Activation and Lung Fibrosis that Conferred by miR-15a Loss in IPF. Cell Death Differ 26 (9), 1832–1844. 10.1038/s41418-018-0250-0 30644438 PMC6748107

[B11] Cheng C. F. Sahu D. Tsen F. Zhao Z. Fan J. Kim R. (2011). A Fragment of Secreted Hsp90α Carries Properties that Enable it to Accelerate Effectively Both Acute and Diabetic Wound Healing in Mice. J. Clin. Invest. 121 (11), 4348–61. 10.1172/JCI46475 22019588 PMC3204835

[B12] Colunga Biancatelli R. M. L. Solopov P. Gregory B. Catravas J. D. (2020). HSP90 Inhibition and Modulation of the Proteome: Therapeutical Implications for Idiopathic Pulmonary Fibrosis (IPF). Int. J. Mol. Sci. 21 (15). 10.3390/ijms21155286 PMC7432830 32722485

[B13] Crookshank J. A. Serrano D. Wang G. S. Patrick C. Morgan B. S. Paré M. F. (2018). Changes in Insulin, Glucagon and ER Stress Precede Immune Activation in Type 1 Diabetes. J. Endocrinol. 239 (2), 181–195. 10.1530/JOE-18-0328 30139929

[B14] Cubillos-Ruiz J. R. Bettigole S. E. Glimcher L. H. (2017). Tumorigenic and Immunosuppressive Effects of Endoplasmic Reticulum Stress in Cancer. Cell 168 (4), 692–706. 10.1016/j.cell.2016.12.004 28187289 PMC5333759

[B15] Dasgupta D. Nakao Y. Mauer A. S. Thompson J. M. Sehrawat T. S. Liao C. Y. (2020). IRE1A Stimulates Hepatocyte-Derived Extracellular Vesicles that Promote Inflammation in Mice with Steatohepatitis. Gastroenterology 159 (4), 1487–e17. e17. 10.1053/j.gastro.2020.06.031 32574624 PMC7666601

[B16] Dong H. Luo L. Zou M. Huang C. Wan X. Hu Y. (2017). Blockade of Extracellular Heat Shock Protein 90α by 1G6-D7 Attenuates Pulmonary Fibrosis through Inhibiting ERK Signaling. Am. J. Physiol. Lung. Cel. Mol. Physiol. 313 (6), L1006–L1015. 10.1152/ajplung.00489.2016 28860147

[B17] Du T. Li H. Fan Y. Yuan L. Guo X. Zhu Q. (2019). The Deubiquitylase OTUD3 Stabilizes GRP78 and Promotes Lung Tumorigenesis. Nat. Commun. 10 (1), 2914. 10.1038/s41467-019-10824-7 31266968 PMC6606649

[B18] Duan F. F. Barron G. Meliton A. Mutlu G. M. Dulin N. O. Schuger L. (2019). P311 Promotes Lung Fibrosis via Stimulation of Transforming Growth Factor-Β1, -β2, and -β3 Translation. Am. J. Respir. Cel Mol. Biol. 60 (2), 221–231. 10.1165/rcmb.2018-0028OC PMC6376409 30230348

[B19] Fan C. S. Chen L. L. Hsu T. A. Chen C. C. Chua K. V. Li C. P. (2019). Endothelial-mesenchymal Transition Harnesses HSP90α-Secreting M2-Macrophages to Exacerbate Pancreatic Ductal Adenocarcinoma. J. Hematol. Oncol. 12 (1), 138. 10.1186/s13045-019-0826-2 31847880 PMC6918594

[B20] Fernandez P. M. Tabbara S. O. Jacobs L. K. Manning F. C. Tsangaris T. N. Schwartz A. M. (2000). Overexpression of the Glucose-Regulated Stress Gene GRP78 in Malignant but Not Benign Human Breast Lesions. Breast Cancer Res. Treat. 59 (1), 15–26. 10.1023/a:1006332011207 10752676

[B21] Guo J. Chang C. Li W. (2017). The Role of Secreted Heat Shock Protein-90 (Hsp90) in Wound Healing - How Could it Shape Future Therapeutics?. Expert Rev. Proteomics 14 (8), 665–675. 10.1080/14789450.2017.1355244 28715921 PMC6557287

[B22] Hosoi T. Nakashima M. Ozawa K. (2018). Incorporation of the Endoplasmic Reticulum Stress-Induced Spliced Form of XBP1 mRNA in the Exosomes. Front. Physiol. 9, 1357. 10.3389/fphys.2018.01357 30319453 PMC6168632

[B23] Hsu H. S. Liu C. C. Lin J. H. Hsu T. W. Hsu J. W. Su K. (2017). Involvement of ER Stress, PI3K/AKT Activation, and Lung Fibroblast Proliferation in Bleomycin-Induced Pulmonary Fibrosis. Sci. Rep. 7 (1), 14272. 10.1038/s41598-017-14612-5 29079731 PMC5660192

[B24] Kakazu E. Mauer A. S. Yin M. Malhi H. (2016a). Hepatocytes Release Ceramide-Enriched Pro-inflammatory Extracellular Vesicles in an IRE1α-dependent Manner. J. Lipid Res. 57 (2), 233–245. 10.1194/jlr.M063412 26621917 PMC4727419

[B25] Kakazu E. Mauer A. S. Yin M. Malhi H. (2016b). Hepatocytes Release Ceramide-Enriched Pro-inflammatory Extracellular Vesicles in an IRE1α-dependent Manner. J. Lipid Res. 57 (2), 233–245. 10.1194/jlr.M063412 26621917 PMC4727419

[B26] Kwon O. C. Lee E. J. Chang E. J. Youn J. Ghang B. Hong S. (2018). IL-17A+GM-CSF+ Neutrophils Are the Major Infiltrating Cells in Interstitial Lung Disease in an Autoimmune Arthritis Model. Front. Immunol. 9, 1544. 10.3389/fimmu.2018.01544 30013577 PMC6036238

[B27] Lee K. I. Su C. C. Fang K. M. Wu C. C. Wu C. T. Chen Y. W. (2020). Ultrafine Silicon Dioxide Nanoparticles Cause Lung Epithelial Cells Apoptosis via Oxidative Stress-Activated PI3K/Akt-Mediated Mitochondria- and Endoplasmic Reticulum Stress-dependent Signaling Pathways. Sci. Rep. 10 (1), 9928. 10.1038/s41598-020-66644-z 32555254 PMC7303152

[B28] Lee T. H. Yeh C. F. Lee Y. T. Shih Y. C. Chen Y. T. Hung C. T. (2020a). Fibroblast-enriched Endoplasmic Reticulum Protein TXNDC5 Promotes Pulmonary Fibrosis by Augmenting TGFβ Signaling through TGFBR1 Stabilization. Nat. Commun. 11 (1), 4254. 10.1038/s41467-020-18047-x 32848143 PMC7449970

[B29] Lee T. H. Yeh C. F. Lee Y. T. Shih Y. C. Chen Y. T. Hung C. T. (2020b). Fibroblast-enriched Endoplasmic Reticulum Protein TXNDC5 Promotes Pulmonary Fibrosis by Augmenting TGFβ Signaling through TGFBR1 Stabilization. Nat. Commun. 11 (1), 4254. 10.1038/s41467-020-18047-x 32848143 PMC7449970

[B30] Li G. Jin F. Du J. He Q. Yang B. Luo P. (2019). Macrophage-secreted TSLP and MMP9 Promote Bleomycin-Induced Pulmonary Fibrosis. Toxicol. Appl. Pharmacol. 366, 10–16. 10.1016/j.taap.2019.01.011 30653976

[B31] Li W. Li Y. Guan S. Fan J. Cheng C. F. Bright A. M. (2007). Extracellular Heat Shock Protein-90alpha: Linking Hypoxia to Skin Cell Motility and Wound Healing. EMBO J. 26 (5), 1221–1233. 10.1038/sj.emboj.7601579 17304217 PMC1817627

[B32] Li W. Sahu D. Tsen F. (2012). Secreted Heat Shock Protein-90 (Hsp90) in Wound Healing and Cancer. Biochim. Biophys. Acta 1823 (3), 730–741. 10.1016/j.bbamcr.2011.09.009 21982864 PMC3266443

[B33] Liu J. Fan L. Yu H. Zhang J. He Y. Feng D. (2019). Endoplasmic Reticulum Stress Causes Liver Cancer Cells to Release Exosomal miR-23a-3p and Up-Regulate Programmed Death Ligand 1 Expression in Macrophages. Hepatology 70 (1), 241–258. 10.1002/hep.30607 30854665 PMC6597282

[B34] Martinez F. J. Collard H. R. Pardo A. Raghu G. Richeldi L. Selman M. (2017). Idiopathic Pulmonary Fibrosis. Nat. Rev. Dis. Primers 3, 17074. 10.1038/nrdp.2017.74 29052582

[B35] Matsuzaki S. Hiratsuka T. Taniguchi M. Shingaki K. Kubo T. Kiya K. (2015). Physiological ER Stress Mediates the Differentiation of Fibroblasts. PLoS One 10 (4), e0123578. 10.1371/journal.pone.0123578 25928708 PMC4416017

[B36] Merkel A. Chen Y. George A. (2019). Endocytic Trafficking of DMP1 and GRP78 Complex Facilitates Osteogenic Differentiation of Human Periodontal Ligament Stem Cells. Front. Physiol. 10, 1175. 10.3389/fphys.2019.01175 31572220 PMC6751249

[B37] Peñaranda-Fajardo N. M. Meijer C. Liang Y. Dijkstra B. M. Aguirre-Gamboa R. den Dunnen W. F. A. (2019). ER Stress and UPR Activation in Glioblastoma: Identification of a Noncanonical PERK Mechanism Regulating GBM Stem Cells through SOX2 Modulation. Cell Death Dis. 10 (10), 690. 10.1038/s41419-019-1934-1 31534165 PMC6751174

[B38] Penke L. R. Speth J. M. Dommeti V. L. White E. S. Bergin I. L. Peters-Golden M. (2018). FOXM1 Is a Critical Driver of Lung Fibroblast Activation and Fibrogenesis. J. Clin. Invest. 128 (6), 2389–2405. 10.1172/JCI87631 29733296 PMC5983327

[B39] Richeldi L. Collard H. R. Jones M. G. (2017). Idiopathic Pulmonary Fibrosis. Lancet 389 (10082), 1941–1952. 10.1016/S0140-6736(17)30866-8 28365056

[B40] Sanchez J. Carter T. R. Cohen M. S. Blagg B. S. J. (2020). Old and New Approaches to Target the Hsp90 Chaperone. Curr. Cancer Drug Targets 20 (4), 253–270. 10.2174/1568009619666191202101330 31793427 PMC7502213

[B41] Shi J. Yu J. Zhang Y. Wu L. Dong S. Wu L. (2019). PI3K/Akt Pathway-Mediated HO-1 Induction Regulates Mitochondrial Quality Control and Attenuates Endotoxin-Induced Acute Lung Injury. Lab. Invest. 99 (12), 1795–1809. 10.1038/s41374-019-0286-x 31570770

[B42] Shuda M. Kondoh N. Imazeki N. Tanaka K. Okada T. Mori K. (2003). Activation of the ATF6, XBP1 and Grp78 Genes in Human Hepatocellular Carcinoma: a Possible Involvement of the ER Stress Pathway in Hepatocarcinogenesis. J. Hepatol. 38 (5), 605–614. 10.1016/s0168-8278(03)00029-1 12713871

[B43] Song M. Bode A. M. Dong Z. Lee M. H. (2019a). AKT as a Therapeutic Target for Cancer. Cancer Res. 79 (6), 1019–1031. 10.1158/0008-5472.CAN-18-2738 30808672

[B44] Song M. Peng H. Guo W. Luo M. Duan W. Chen P. (2019b). Cigarette Smoke Extract Promotes Human Lung Myofibroblast Differentiation by the Induction of Endoplasmic Reticulum Stress. Respiration 98 (4), 347–356. 10.1159/000502099 31416082

[B45] Tanimura A. Miyoshi K. Horiguchi T. Hagita H. Fujisawa K. Noma T. (2018). Mitochondrial Activity and Unfolded Protein Response Are Required for Neutrophil Differentiation. Cell. Physiol. Biochem. 47 (5), 1936–1950. 10.1159/000491464 29972819

[B46] Trepel J. Mollapour M. Giaccone G. Neckers L. (2010). Targeting the Dynamic HSP90 Complex in Cancer. Nat. Rev. Cancer 10 (8), 537–549. 10.1038/nrc2887 20651736 PMC6778733

[B47] van Lidth de Jeude J. F. Spaan C. N. Meijer B. J. Smit W. L. Soeratram T. T. D. Wielenga M. C. B. (2018). Heterozygosity of Chaperone Grp78 Reduces Intestinal Stem Cell Regeneration Potential and Protects against Adenoma Formation. Cancer Res. 78 (21), 6098–6106. 10.1158/0008-5472.CAN-17-3600 30232220 PMC8272399

[B48] Wan H. Xie T. Xu Q. Hu X. Xing S. Yang H. (2019). Thy-1 Depletion and Integrin β3 Upregulation-Mediated PI3K-Akt-mTOR Pathway Activation Inhibits Lung Fibroblast Autophagy in Lipopolysaccharide-Induced Pulmonary Fibrosis. Lab. Invest. 99 (11), 1636–1649. 10.1038/s41374-019-0281-2 31249375 PMC7102294

[B49] Wang H. Yu Z. Huo S. Chen Z. Ou Z. Mai J. (2018). Overexpression of ELF3 Facilitates Cell Growth and Metastasis through PI3K/Akt and ERK Signaling Pathways in Non-small Cell Lung Cancer. Int. J. Biochem. Cel. Biol. 94, 98–106. 10.1016/j.biocel.2017.12.002 29208568

[B50] Wang Y. Lin Y. Wang L. Zhan H. Luo X. Zeng Y. (2020). TREM2 Ameliorates Neuroinflammatory Response and Cognitive Impairment via PI3K/AKT/FoxO3a Signaling Pathway in Alzheimer's Disease Mice. Aging (Albany NY) 12, 20862–20879. 10.18632/aging.104104 33065553 PMC7655179

[B51] Wolters P. J. Collard H. R. Jones K. D. (2014). Pathogenesis of Idiopathic Pulmonary Fibrosis. Annu. Rev. Pathol. 9, 157–179. 10.1146/annurev-pathol-012513-104706 24050627 PMC4116429

[B52] Xu W. Wu Y. Hu Z. Sun L. Dou G. Zhang Z. (2019). Exosomes from Microglia Attenuate Photoreceptor Injury and Neovascularization in an Animal Model of Retinopathy of Prematurity. Mol. Ther. Nucleic Acids 16, 778–790. 10.1016/j.omtn.2019.04.029 31163320 PMC6545376

[B53] Yao L. Zhao H. Tang H. Liang J. Liu L. Dong H. (2016). The Receptor for Advanced Glycation End Products Is Required for β-catenin Stabilization in a Chemical-Induced Asthma Model. Br. J. Pharmacol. 173 (17), 2600–2613. 10.1111/bph.13539 27332707 PMC4978160

[B54] Yao X. Tu Y. Xu Y. Guo Y. Yao F. Zhang X. (2020). Endoplasmic Reticulum Stress-Induced Exosomal miR-27a-3p Promotes Immune Escape in Breast Cancer via Regulating PD-L1 Expression in Macrophages. J. Cel. Mol. Med. 24 (17), 9560–9573. 10.1111/jcmm.15367 PMC7520328 32672418

[B55] Yoshida H. (2007). ER Stress and Diseases. FEBS J. 274 (3), 630–658. 10.1111/j.1742-4658.2007.05639.x 17288551

[B56] Zhang G. Liu Z. Ding H. Zhou Y. Doan H. A. Sin K. W. T. (2017). Tumor Induces Muscle Wasting in Mice through Releasing Extracellular Hsp70 and Hsp90. Nat. Commun. 8 (1), 589. 10.1038/s41467-017-00726-x 28928431 PMC5605540

[B57] Zhou X. Wen Y. Tian Y. He M. Ke X. Huang Z. (2019). Heat Shock Protein 90α-dependent B-Cell-2-Associated Transcription Factor 1 Promotes Hepatocellular Carcinoma Proliferation by Regulating MYC Proto-Oncogene C-MYC mRNA Stability. Hepatology 69 (4), 1564–1581. 10.1002/hep.30172 30015413 PMC6586158

