# Corrigendum: Extracellular HSP90α interacts with ER stress to promote fibroblasts activation through PI3K/AKT pathway in pulmonary fibrosis

**DOI:** 10.3389/fphar.2024.1296214

**Published:** 2024-08-12

**Authors:** Jinming Zhang, Wenshan Zhong, Yuanyuan Liu, Weimou Chen, Ye Lu, Zhaojin Zeng, Yujie Qiao, Haohua Huang, Xuan Wan, Wei Li, Xiaojing Meng, Fei Zou, Shaoxi Cai, Hangming Dong

**Affiliations:** ^1^ Chronic Airways Diseases Laboratory, Department of Respiratory and Critical Care Medicine, Nanfang Hospital, Southern Medical University, Guangzhou, China; ^2^ Department of Dermatology and The Norris Comprehensive Cancer Centre, University of Southern California Keck Medical Centre, Los Angeles, CA, United States; ^3^ School of Public Health, Southern Medical University, Guangzhou, China

**Keywords:** extracellular Hsp90α, er stress, fibroblasts activation, PI3K/AKT, pulmonary fibrosis

In the published article, there were two errors in Figure 3B, D as published. The Masson staining picture in the TUDCA group was misplaced in Figure 3B. Additionally, the Western blot picture of GRP78 was misplaced in Figure 3D. After checking the raw data, the misplaced Masson staining picture of TUDCA group in Figure 3B and the Western blot picture of GRP78 in Figure 3D were corrected. The corrected Figure 3 and its caption appears below.

**FIGURE 3:**
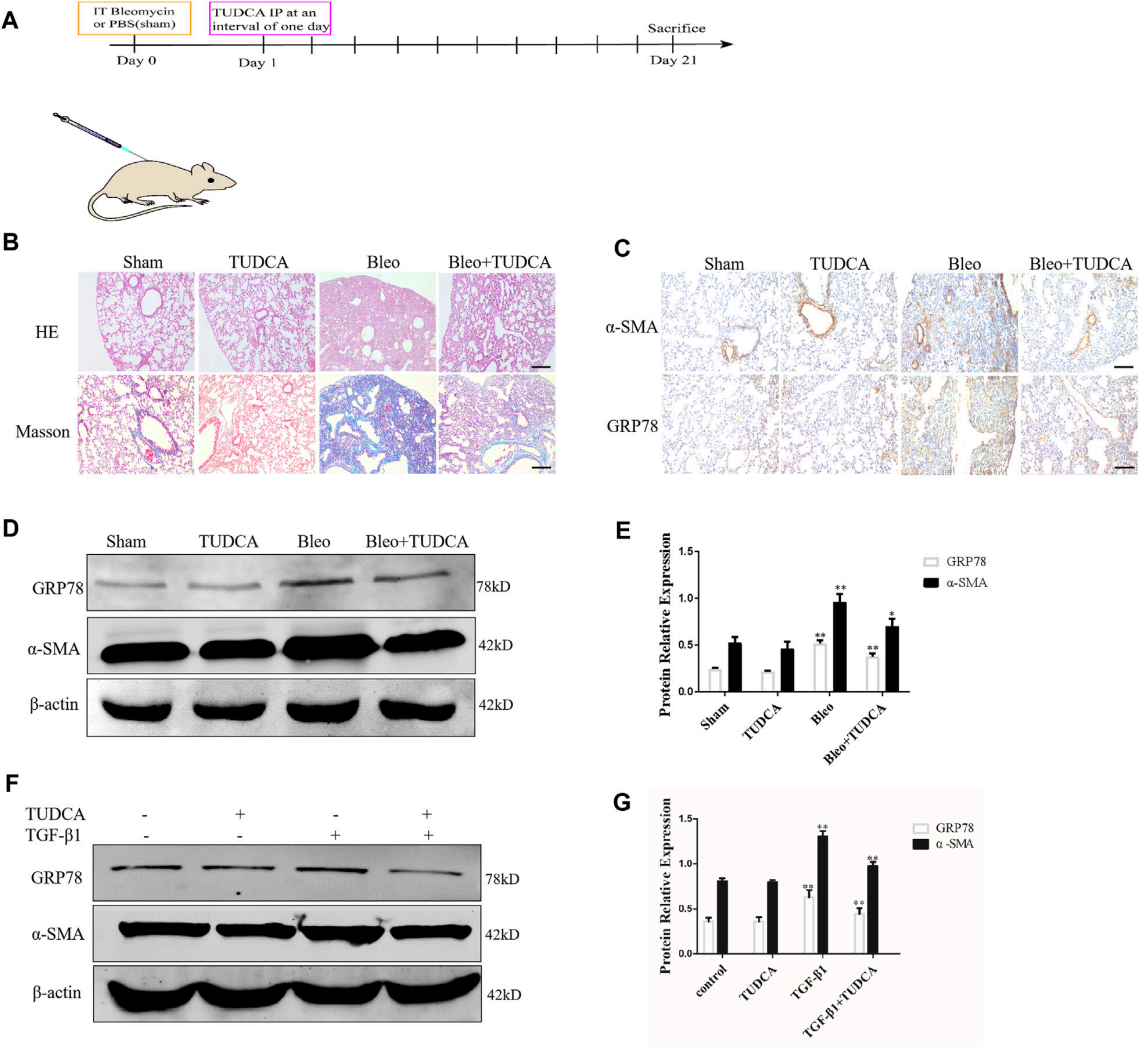
ER stress in the lung fibroblasts is critical for pulmonary fibrosis progression. **(A)** Experimental scheme of the mouse model of bleomycin-induced pulmonary fibrosis. Mice were intratracheally injected with saline or bleomycin (3 mg/kg) at day 0. On day 1, mice were administrated with TUDCA (50 mg/kg) or DMSO by intraperitoneal injection every 2 days. Mice were sacrificed on day 21 (*n* = 10 for each group). **(B)** Histological images and collagen deposition of the lung tissue was detected by H&E and Masson staining. Scale bar = 100 μm. **(C)** Representative images showing GRP78 and α-SMA staining of lung tissues of mice treated with saline, bleomycin without or with TUDCA. Scale bar = 100 μm. **(D, E)** Western blot analysis of expression of GRP78 and α -SMA. **(F, G)**, IMR90 were pre-treated with TUDCA (100 μM) for 2 h and followed by TGF-β1 (10 ng/mL) for 24 h. The expression levels of GRP78 and α-SMA was measured by Western blot. β-actin was used as an internal control. *p < 0.05, **p < 0.01.

In the published article, there was an error in Figure 7B. The IHC staining picture was misplaced in Figure 7B. After checking the raw data, the misplaced IHC staining picture in Figure 7B was corrected. The corrected Figure 7 and its caption appears below.

**FIGURE 7:**
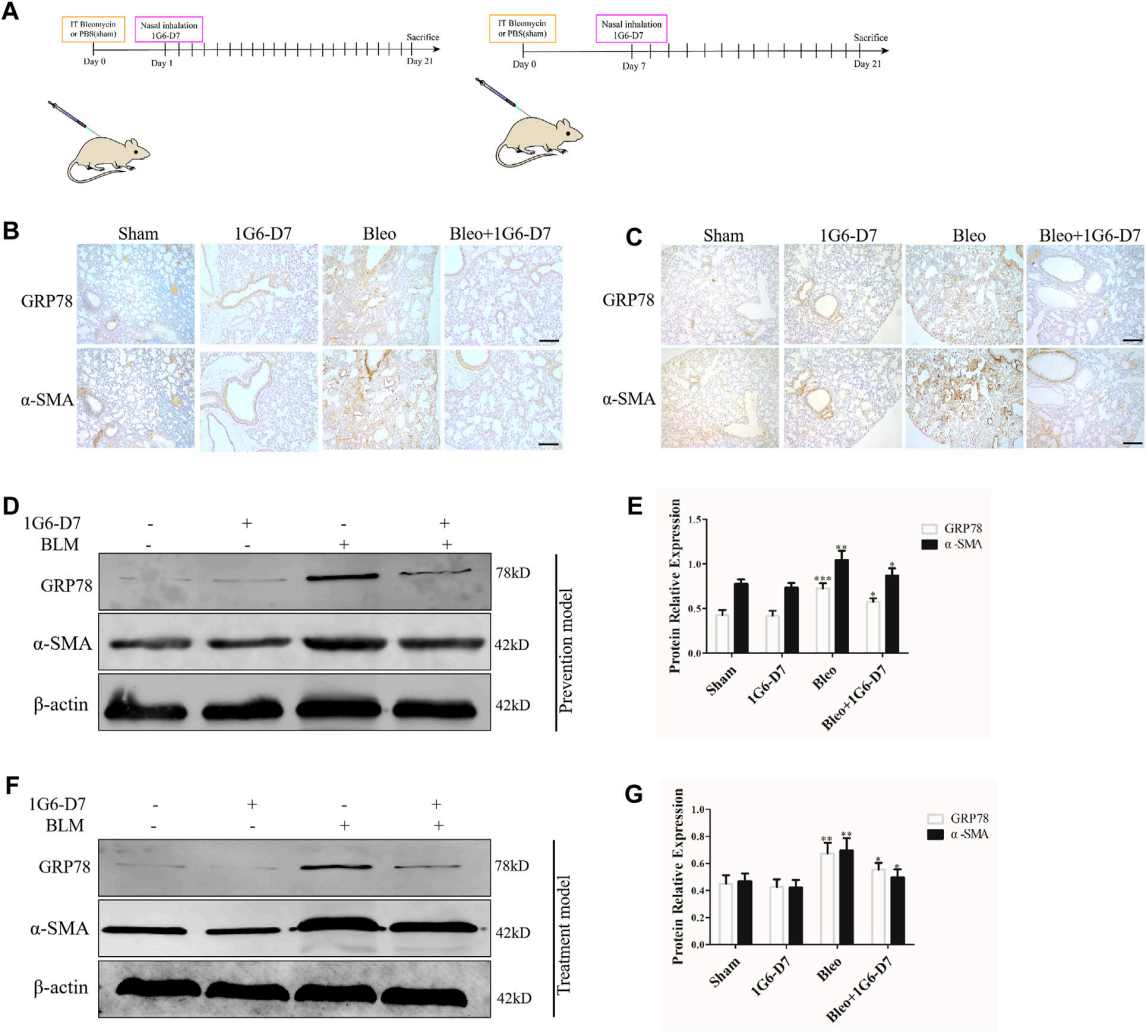
Monoclonal antibody 1G6-D7 inhibits ER stress in the bleomycin-induced pulmonary fibrosis model. **(A)** Schematic diagram of mouse model establishment (n = 10 for each group). **(B)** Representative images showing GRP78 and α-SMA staining of lung tissues of mice in the prophylactical model. Scale bar = 100 μm. **(C)** Representative images showing GRP78 and α-SMA staining of lung tissues of mice in the therapeutical model. Scale bar = 100 μm. Western blot analysis of the expression of GRP78 and α-SMA in the prophylactical model **(D, E)** and therapeutical model **(F, G)**. β-actin was used as an internal control. *p < 0.05, **p < 0.01.

The authors apologize for these errors and state that this does not change the scientific conclusions of the article in any way. The original article has been updated.

